# First-in-Human Experience With the EnCompass F_2_ Filter, a Novel Cerebral Embolic Protection Device for TAVR

**DOI:** 10.1016/j.jscai.2025.103608

**Published:** 2025-08-12

**Authors:** Isaac George, David Blusztein, Irakli Gogorishvili, Gvantsa Dughashvili, May Nour, Viktor Szeder, Keith Woodward, Tamim M. Nazif

**Affiliations:** a Structural Heart and Valve Center, Columbia University Irving Medical Center, New York, New York; b Division of Cardiology, Victorian Heart Hospital, Monash Health, Melbourne, Victoria, Australia; c Department of Interventional Cardiology, Israeli-Georgian Medical Research Clinic, Tbilisi, Georgia; d eHealth Merit Corporation, Los Angeles, California; e Vista Radiology and Oculus Imaging, Knoxville, Tennessee

**Keywords:** cerebral embolic protection, stroke, transcatheter aortic valve replacement

## Abstract

**Background:**

Stroke is a feared complication of transcatheter aortic valve replacement (TAVR), and effective cerebral embolic protection devices are an important innovation target. The EnCompass F_2_ system is a novel cerebral embolic protection device consisting of a cylindrical, nitinol frame and an electrospun polyurethane deflection filter with 30-μm average pore size. It is deployed across the aortic arch from femoral access to provide complete cerebral embolic protection during TAVR.

**Methods:**

This first-in-human study investigated the feasibility and safety of F_2_ filter use during TAVR. Subjects had symptomatic severe aortic stenosis and met established clinical indications for TAVR. The primary safety end point was 30-day major adverse cardiac and cerebrovascular events, defined as all-cause death, stroke, major vascular complication, type 2 to 4 bleeding, or acute kidney injury stage 3 to 4. F_2_ filter technical and procedure success and new ischemic brain lesion counts and volumes on diffusion-weighted magnetic resonance imaging (DW-MRI) were evaluated.

**Results:**

Twelve patients underwent transfemoral TAVR with the F_2_ device. Subjects were 58% female with mean age 73.4 ± 5.1 years and mean Society of Thoracic Surgeons score 3.2 ± 2.0%. A balloon-expandable valve was used in 75% (n = 9). A single F_2_ device was used in all cases and was delivered ipsilateral to the TAVR sheath in 41.7% (n = 5). Technical and procedure success was achieved in 100% of cases. No major adverse cardiac and cerebrovascular events occurred within 30 days of TAVR, including no strokes. DW-MRI revealed median total new lesion volume 23.4 mm^3^ (IQR: 13.1-159.8 mm^3^).

**Conclusions:**

In this first-in-human series, cerebral embolic protection with the EnCompass F_2_ during TAVR was feasible and safe with very low new brain DW-MRI lesion volumes and no strokes.

## Introduction

Transcatheter aortic valve replacement (TAVR) has revolutionized the treatment of aortic stenosis with an excellent safety profile overall. However, stroke was identified early in the TAVR experience as an important procedural complication that was associated with significant morbidity and mortality.^1^, ^2^, ^3^ Despite improvements in procedural techniques and device technology, periprocedural stroke continues to occur in up to 3% of TAVR cases in contemporary practice.^4^^,^^5^ The majority of stroke after TAVR occurs within the immediate periprocedural period (<72 hours) and is understood to be due to the liberation of embolic debris during TAVR device delivery across the aortic arch and valve deployment or other manipulations.^3^^,^^6^ Beyond clinical stroke, diffusion-weighted magnetic resonance imaging (DW-MRI) studies have demonstrated ischemic brain injury in the vast majority of TAVR cases (68%-93%), with uncertain long-term clinical consequences.^7^^,^^8^

**Figure 1:**
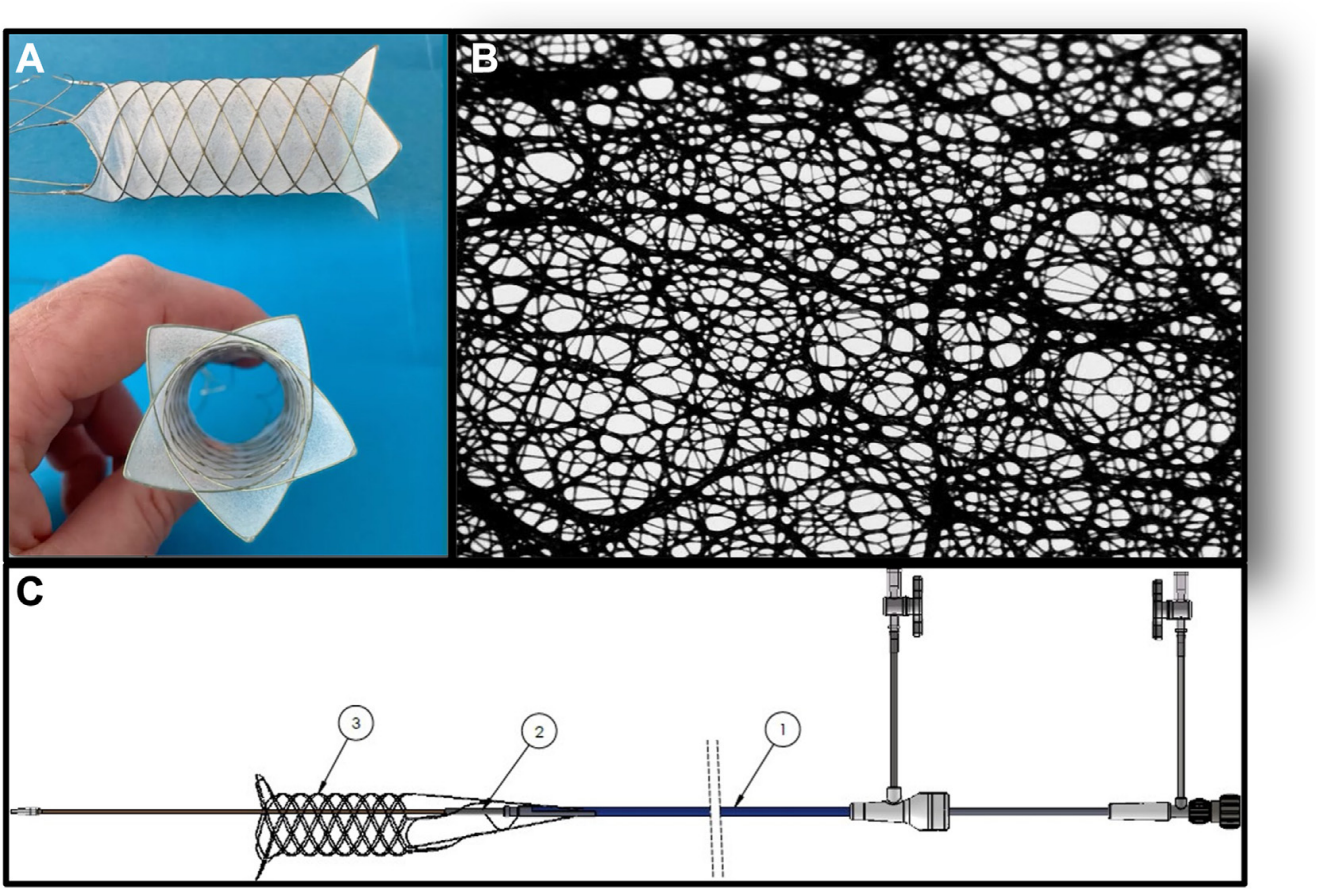
**EnCompass F_2_ filter.** (**A**) The F_2_ filter is comprised of a self-expanding nitinol frame and an electrospun polyurethane filter. (**B**) Microscopic appearance of the electrospun polyurethane filter demonstrating the distribution of pore sizes, averaging 30 μm and with maximum pore size of 80 μm. (**C**) The system is comprised of (1) *F*_*2*_*delivery sheath* for filter delivery and retrieval, (2) *F*_*2*_*pusher assembly and 0.015” retention wire* to facilitate filter placement and removal, and (3) *F*_*2*_*filter*.

Safe and effective cerebral embolic protection devices (CEPD) to mitigate the risk of embolic brain injury and stroke during TAVR remain an unmet clinical need. A number of CEPDs have been developed with varying filter designs and deployment techniques. The most widely available device is a partial intravascular filter with 150-μm pore size that is delivered by radial artery access and has been shown in randomized trials to be safe but has not demonstrated clinical efficacy in preventing stroke.^4^^,^^9^ The EnCompass F_2_ system (EnCompass Technologies) is a novel CEPD that consists of a hollow, cylindrical, nitinol frame covered by an electrospun polyurethane filter with an average pore size of 30 μm (Figure 1). It is delivered by ipsilateral or contralateral femoral artery access and isolates the aortic arch, deflecting embolic debris away from the brain circulation during TAVR. This circumferential arch deflector design provides complete cerebral protection with stable device anchoring in the aorta and a much smaller filter pore size than previously available.

The current report consists of a first-in-human (FIH) series of cases of embolic protection with the EnCompass F_2_ device during transfemoral TAVR.

## Methods

This FIH series evaluated the safety and feasibility of cerebral embolic protection with the EnCompass F_2_ device during transfemoral TAVR. All subjects met established clinical indications for TAVR for symptomatic severe, native aortic valve stenosis. Important exclusion criteria included a history of stroke or transient ischemic attack within 6 months, known contraindication to MRI, and unsuitable aortic arch or iliofemoral arterial anatomy, including heavy calcification, severe atheroma, or severe tortuosity, as assessed by computed tomography angiography (Supplemental Table S1). This FIH study recruited patients (nonconsecutive) meeting protocol-defined eligibility criteria from a single center at the Israeli-Georgian Medical Research Clinic in Tbilisi, Georgia between April 2023 and May 2024. A single team of trained operators (T.N., I.G., I.G.) performed all procedures in the current series. The study protocol was approved by the local institutional ethics committee and informed consent was obtained from all subjects.

### Study device

The EnCompass F_2_ device is an arch deflector CEPD consisting of a superelastic, braided nitinol frame that supports an electrospun polyurethane filter membrane (Figure 1A). It is a self-expanding, cylindrical device that is delivered to the aortic arch by means of a 13.5F delivery system and expands to achieve 360° wall apposition for stability. The frame is connected to a 0.015-inch nitinol wire tether that remains externalized at the femoral access site and is ultimately used to recapture and remove the device at the conclusion of the procedure. While in place, the F_2_ device completely filters all blood entering the three great vessels feeding the cerebral circulation, without requiring access into these vessels, and deflects debris away from the brain. The electrospun filter is constructed with a distribution of pore sizes, averaging 30 μm with a maximum pore size of 80 μm (Figure 1B).

### Study procedure

#### Femoral artery access

Bilateral femoral artery access is obtained in the standard fashion, and the access sites are preclosed with suture-mediated closure devices if desired. The F_2_ filter and delivery system (13.5F) can then be delivered through a 14F sheath contralateral to the TAVR sheath or ipsilaterally through the TAVR sheath access site. After filter deployment, a 0.015-inch retention wire remains at the access site to facilitate postprocedural device recovery. In the case of contralateral filter placement, the angiographic pigtail catheter can be placed through the same sheath as the retention wire. For ipsilateral filter access, a second 0.035-inch guide wire is placed in the 14F sheath alongside the retention wire, the sheath is removed, and the TAVR sheath is readvanced over the new wire such that the retention wire is externalized outside of the TAVR sheath. In this case, a standard 5F sheath can be used contralaterally for the pigtail catheter.

#### F_2_ filter deployment

**Figure 2:**
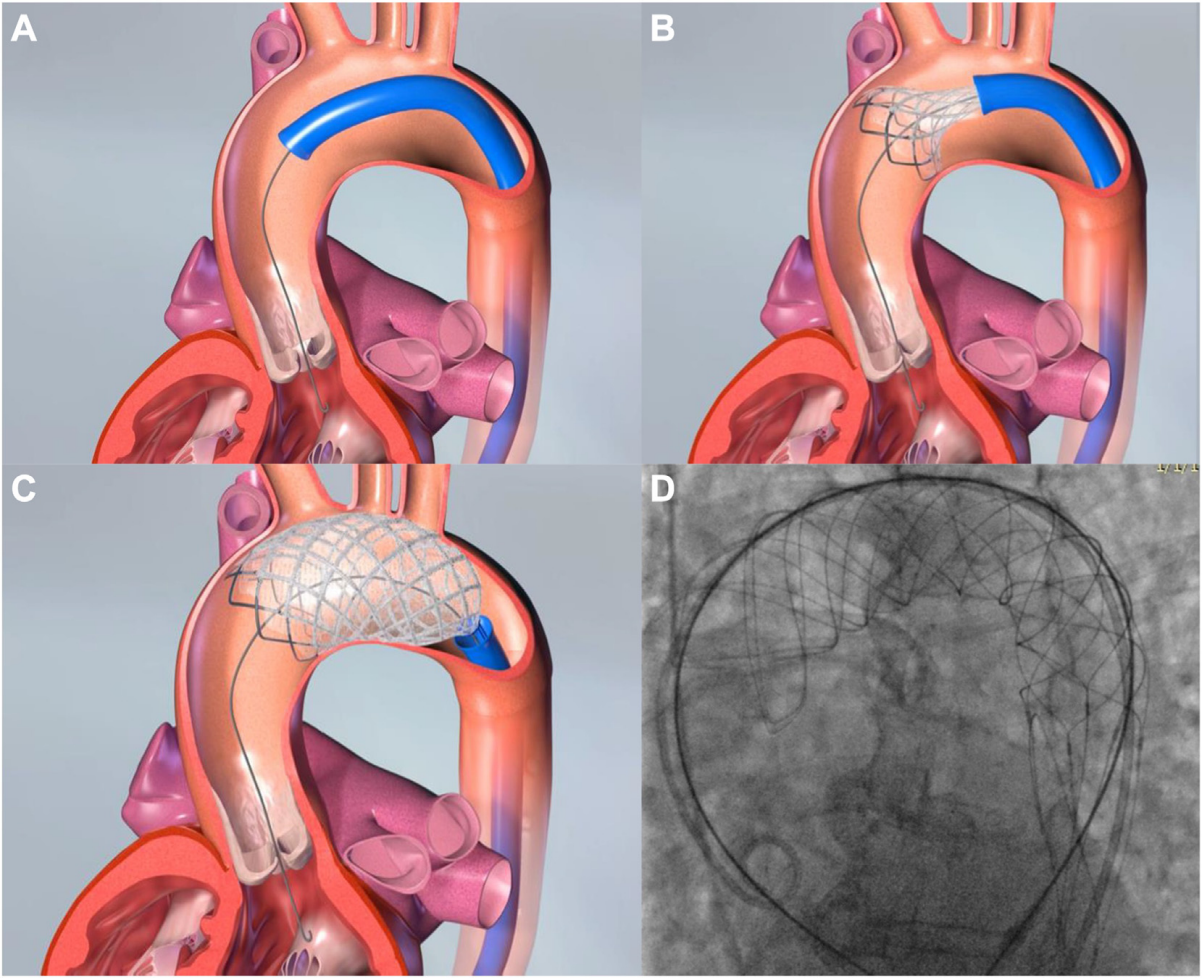
**F_2_ filter delivery.** F_2_ filter deployment is demonstrated. (**A**) Delivery sheath is positioned proximal to the brachiocephalic artery origin. (**B**) The filter is withdrawn from the delivery sheath with a pin and pull technique. (**C**) The filter's distal portion is covering the left subclavian artery origin. (**D**) A fluoroscopic appearance of the final position of the filter, which is stabilized via its radial force within the aorta.

After appropriate preparation and flushing, the F_2_ filter and delivery sheath are advanced across the aortic arch over a stiff guide wire. An aortic angiogram is performed to locate the origin of the head and neck vessels and to select a proximal landing zone approximately 2 cm from the takeoff of the innominate artery. The F_2_ filter is then unsheathed and deployed using a pin (F_2_ pusher) and pull (F_2_ delivery sheath) technique (Figure 2B, C). The delivery sheath is withdrawn over both the guide wire and retention wire, and the F_2_ filter remains stabilized at the deployment site via radial force (Figure 2D).

#### TAVR procedure

**Figure 3:**
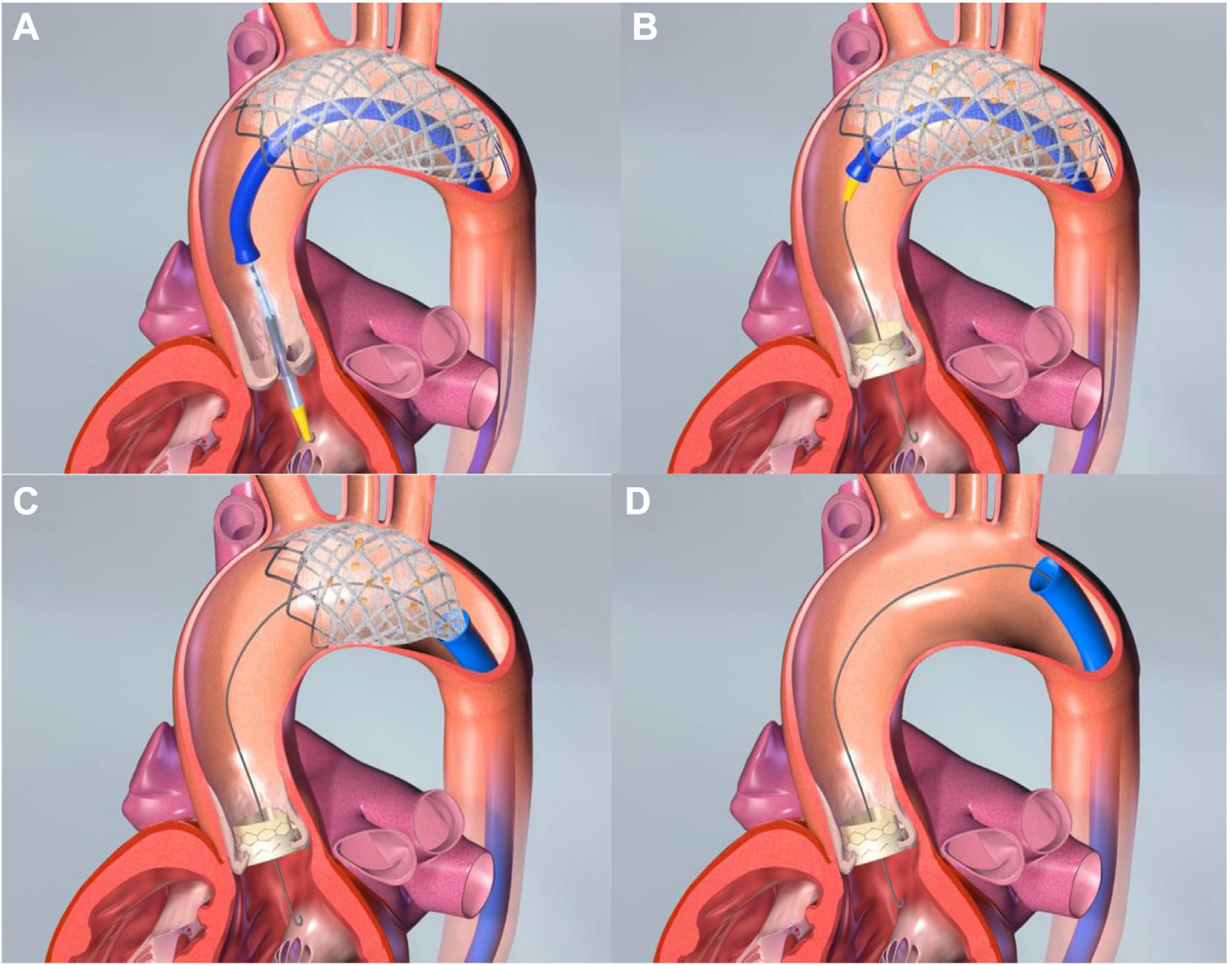
**TAVR deployment and F_2_ filter retrieval.** (**A** and **B**) The TAVR procedure is performed through the central lumen of the F_2_ filter. (**C** and **D**) Then the F_2_ filter is retrieved via resheathing. TAVR, transcatheter aortic valve replacement.

At this stage, the F_2_ filter device is recrossed from the other femoral access site with a pigtail catheter and guide wire such that there are 2 guide wires in the aortic root to accommodate the angiographic pigtail catheter and the TAVR device. The aortic valve is then crossed and the transfemoral TAVR is performed in the usual fashion. The F_2_ filter remains in-place throughout the TAVR procedure, including any preceding or subsequent balloon valvuloplasty, which is also performed through the central lumen of the F_2_ filter (Figure 3A, B).

#### F2 filter recovery

After the completion of the TAVR procedure and removal of the TAVR delivery system and the pigtail catheter, the F_2_ sheath is reinserted over the filter retention wire and advanced to the filter location (Figure 3C, D). While holding the retention wire, the F_2_ delivery sheath is advanced over the filter until it is completely recaptured, at which point the entire system is carefully removed through the access sheath. In the case of ipsilateral access, this requires removing the TAVR sheath and readvancing a 14F sheath over the retention wire and an additional parallel 0.018-inch wire, such that the retention wire is again inside the sheath. After F_2_ filter removal, hemostasis is then achieved in the typical fashion.

### Study end points

The primary safety end point was 30-day major adverse cardiac and cerebrovascular event (MACCE), defined as all-cause death, all stroke (nondisabling and disabling), major vascular complications, type 2 to 4 bleeding, or stage 3 or 4 acute kidney injury (7 days). The individual safety component end points were also examined. Device performance end points included technical success, defined as successful F_2_ filter deployment, stable device positioning, complete coverage of the head and neck vessels during TAVR, and successful filter retrieval. Procedural success was defined as technical success in the absence of any F_2_-related or F_2_ procedure-related adverse safety events. DW-MRI of the brain was performed at 8 to 72 hours post procedure (preferably within 24 hours) to assess for total new brain lesion volume, median individual new lesion volume, and median number of new lesions. Remote neurocognitive testing was performed at discharge or 5 to 10 days, whichever occurred first. Clinical events were adjudicated by an independent clinical events committee, and MRI end points were measured by an independent core laboratory (Oculus Imaging). Remote neurological examination was performed by telehealth through a clinical core laboratory (blinded, independent vascular neurologists; eHealth Merit Corporation). The study was approved by the Georgian Ministry of Health and the local ethics committee, the Israeli-Georgian Medical Research Clinic Healthycore Ltd.

## Results

Twelve patients underwent transfemoral TAVR with cerebral embolic protection with the F_2_ device. The mean age was 73.4 ± 5.1 years, the mean Society of Thoracic Surgeons (STS) score was 3.2 ± 2.0, and 58% were female (n = 7) (Table 1). Baseline comorbidities included prior transient ischemic attack (n = 1), atrial fibrillation (n = 1), significant carotid artery disease (n = 1), diabetes (n = 3), and obesity with body mass index >30 kg/m^2^ (n = 5). Procedural data are described in Table 2. TAVR was performed with a balloon-expandable valve in 75% (n = 9), and the F_2_ filter was deployed from contralateral femoral access in the first 7 cases and ipsilateral access in the final 5 cases. A single filter was used in all cases with filter repositioning before TAVR in a single case, and the mean deployment time was 3.3 ± 5.5 minutes. Device technical and procedural success were achieved in 100% of cases, and there were no adverse events related to the F_2_ filter or F_2_ filter procedure.

**Table 1:** Baseline characteristics.

Characteristic	N = 12
Female sex	58.3% (7)
Age, y	73.4 ± 5.07
Body mass index, kg/m^2^	29.1 ± 3.56
Creatinine, mg/dL	0.9 ± 0.23
Prior stroke	0% (0)
Prior transient ischemic attack	8.3% (1)
Atrial fibrillation/atrial flutter	8.3% (1)
Pacemaker or defibrillator	0% (0)
Hypertension	100% (12)
Aortic disease (aneurysm)	0% (0)
Carotid artery disease	8.3% (1)
Peripheral vascular disease	0% (0)
Chronic kidney disease	0% (0)
Congestive heart failure	50.0% (6)
Chronic lung disease	8.3% (1)
Diabetes	25.0% (3)

Values are % (n) or mean ± SD.

**Table 2:** TAVR procedure information.

Characteristics	N = 12
THV type	
Edwards	75.0% (9)
Medtronic	25.0% (3)
THV size, mm	
20	0% (0)
23	41.7% (5)
26	41.7% (5)
29	16.7% (2)
34	0% (0)
Technical success^a^	100% (12)
No. of EnCompass devices utilized	
1	100.0% (12)
>1	0% (0)
No. of attempts needed to deploy F_2_ filter	
Mean ± SD (N)	1.1 ± 0.29 (12)
Median (min, max)	1.0 (1.0, 2.0)
EnCompass time for placement, min	
Mean ± SD (N)	3.3 ± 5.49 (12)
Median (Min, Max)	1.5 (0.4, 20.3)
Overall TAVR procedure time, min	
Mean ± SD (N)	79.3 ± 26.20 (12)
Median (Min, Max)	71.0 (42.0, 115.0)

Values are % (n) unless otherwise indicated.

TAVR, transcatheter aortic valve replacement; THV, transcatheter heart valve.

a Technical success is defined as successful device deployment, stable device positioning, complete coverage during TAVR, and successful retrieval.

Clinical and imaging outcomes are summarized in Table 3. No MACCE were reported within 30 days of TAVR, including no deaths or strokes. DW-MRI within 72 hours of TAVR revealed a median new lesion count of 2 (IQR, 1-5). The median single new lesion volume was 14.1 mm^3^ (IQR,10.0-34.6 mm^3^), and the median total new lesion volume was 23.4 mm^3^ (IQR, 13.1-159.8 mm^3^). More than half of patients (58%, n = 7) had <50 mm^3^ total new lesion volume, and 91.7% (n = 11) had <500 mm^3^ total new lesion volume. Neurocognitive assessments performed at baseline and at 30 days post TAVR were not significantly different; the mean modified Rankin Scale change from baseline was −0.17 ± 0.39, the mean National Institutes of Health Stroke Scale change from baseline was 0.08 ± 0.29, and the mean Montreal Cognitive Assessment change from baseline was 2.08 ± 3.42.

**Table 3:** Study outcomes.

	N = 12
MACCE (30 d post-procedure)^a^	0% (0)
DW-MRI average new lesion count	
Mean ± SD (N)	4 ± 5.5 (12)
Median (Min, Max)	1 (0, 20)
DW-MRI average single new lesion volume, mm^3^	
Mean ± SD (N)	31.0 ± 36.6 (12)
Median (Min, Max)	14.1 (0, 150.0)
DW-MRI total new lesion volume, mm^3^	
Mean ± SD (N)	126.4 ± 200.2 (12)
Median (Min, Max)	23.4 (0, 720.0)
mRS change from baseline to 30 d	
Mean ± SD (N)	−0.17 ± 0.39 (12)
(Min, Max) (N)	(−1.00, 0.00) (12)
NIHSS change from baseline to 30 d	
Mean ± SD (N)	0.08 ± 0.29 (12)
(Min, Max) (N)	(0.00, 1.00) (12)
Montreal Cognitive Assessment change from baseline to 30 d	
Mean ± SD (N)	2.08 ± 3.42 (12)
(Min, Max) (N)	(−5.00, 7.00) (12)

Values are % (n) unless otherwise indicated.

MACCE, major adverse cardiac and cerebrovascular events; DW-MRI, diffusion-weighted magnetic resonance imaging; mRS, modified Rankin scale; NIHSS, National Institutes of Health Stroke Scale.

a MACCE is defined as all-cause death, all stroke, major vascular complications, type 2 to 4 bleeding, or acute kidney injury stage 3 or 4 within 7 days.

**Figure 4:**
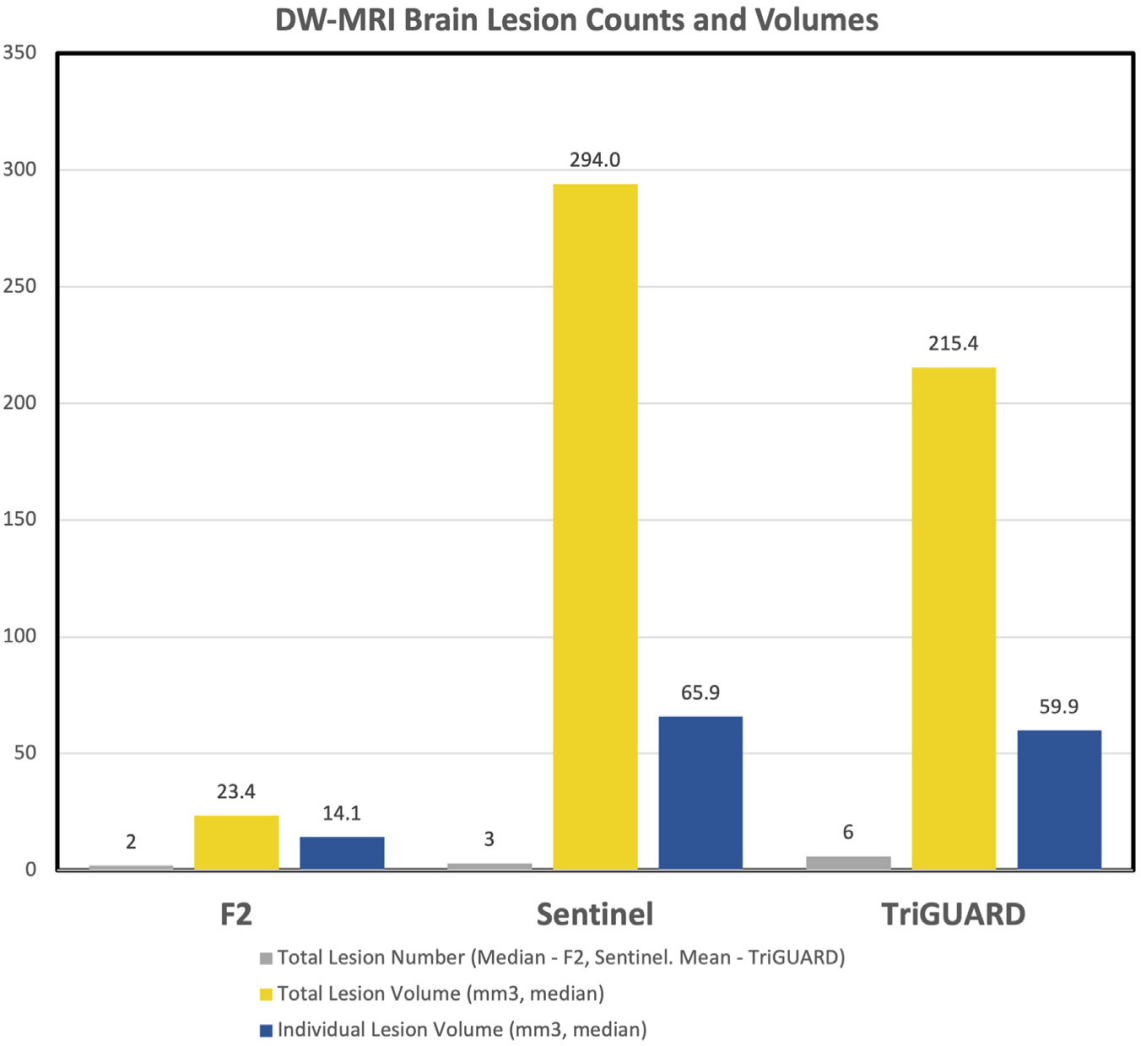
**F_2_ DW-MRI results compared with historical data from SENTINEL and TriGUARD.** Graphic demonstration comparing total lesion number, volume and individual lesion volume in the F_2_ with historical SENTINEL^9^ and TriGUARD^10^ studies. DW-MRI, diffusion-weighted magnetic resonance imaging.

Figure 4 demonstrates a comparison of brain DW-MRI data points from this F_2_ study against historical studies using the SENTINEL and TriGUARD devices.^9^^,^^10^ When compared to SENTINEL and TriGUARD, respectively, F_2_ patients had the lowest total new lesion number (2 vs 3 vs 6), median total lesion volume (23.4 mm^3^ vs 294.0 mm^3^ vs 215.4 mm^3^), and median individual lesion volume (14.1 mm^3^ vs 65.9 mm^3^ vs 59.9 mm^3^).

## Discussion

The EnCompass F_2_ is a novel CEPD that functions as an aortic arch deflector and provides complete cerebral arterial protection with a cylindrical nitinol frame and an electrospun filter of very small pore size (30 μm average). This FIH study of the F_2_ filter during TAVR demonstrates procedural feasibility with an excellent safety profile and very low DW-MRI cerebral ischemic lesion numbers and volumes compared with historical controls (Central Illustration).

**Central Illustration:**
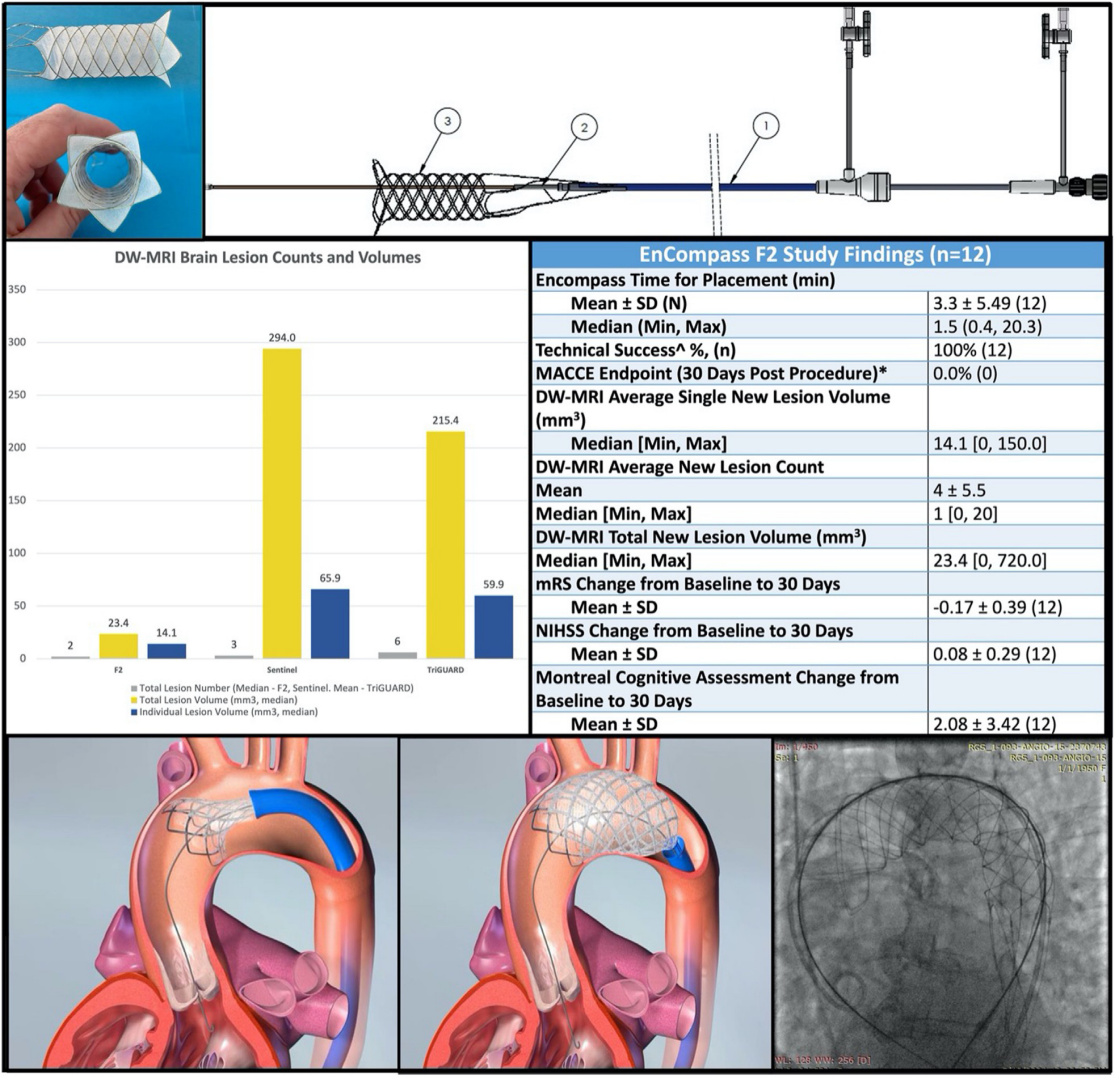
**EnCompass F_2_ embolic filter device and FIH study findings.** DW-MRI, diffusion-weighted magnetic resonance imaging; MACCE, major adverse cardiac and cerebrovascular event; mRS, modified Rankin scale; NIHSS, National Institutes of Health Stroke Scale.

Embolic stroke is an important complication of TAVR with an incidence of 2% to 3% in contemporary practice.^4^^,^^5^ Furthermore, even in the absence of stroke, DW-MRI analyses reveal cerebral ischemic injury from embolic debris in the majority (>70%) of TAVR recipients. Safe and effective CEPD therefore remains an unmet clinical need and an important target of ongoing research. Several CEPDs of varying design concepts, including selective intravascular filters, aortic capture and removal devices, and aortic arch deflectors, have entered or completed clinical trials.

The most widely available CEPD, the SENTINEL Cerebral Protection System (Boston Scientific), is a partial intravascular filter with a 150-μm pore size that is delivered via the right radial artery and deployed in the innominate and left carotid arteries. Clinical trials have shown that the device is safe to use and captures embolic debris in 99% of cases, but have failed to convincingly demonstrate efficacy in reducing stroke or MRI lesions.^4^^,^^9^^,^^11^^,^^12^ The largest of these randomized trial, PROTECTED-TAVR,^4^ included 3000 patients and failed to show a significant difference in the primary end point of all stroke, but did suggest a reduction in the secondary end point of disabling stroke with CEPD compared to control (0.5% vs 1.3%; 95% CI, −1.5 to −0.1). The BHF PROTECT-TAVI trial, a similar randomized trial in the United Kingdom, is nearing completion with enrollment of almost 8000 patients.^13^ However, the continued failure of these trials to meet the primary efficacy end points have raised the question of whether alternative CEPD designs might be more effective.

Newer CEPDs under development include aortic arch-based devices that provide complete cerebral embolic protection either through debris capture and removal or via deflection of debris away from the cerebral circulation. Broadly speaking, capture and removal devices are more complex device systems that may require larger bore femoral access or pose a higher risk of device interactions with TAVR systems. Arch deflectors, on the other hand, tend to be simpler with a lower device profile, but may be vulnerable to difficulty in positioning or device instability. The TriGUARD (Keystone Heart) series of CEPD were the first aortic arch deflector devices to complete randomized clinical trials. The TriGUARD 3 is a self-stabilizing, noncircumferential arch deflector with a polyetheretherketone mesh (pore size 115 × 145 μm). The TriGUARD 3 was evaluated against unprotected TAVR in the randomized REFLECT II trial but failed to meet the primary efficacy end point, which consisted of a hierarchical composite of clinical and DW-MRI end points.^10^ This was potentially related to difficulty in achieving and maintaining complete cerebral protection due to device instability and interactions, and in a post hoc MRI analysis of cases in which the TriGUARD 3 remained in the intended location throughout the procedure (54.3%), there were numeric reductions in total lesion volumes above incremental thresholds.

The F_2_ filter has key design features intended to overcome many of the limitations of prior CEPD, including existing aortic arch deflectors. First, the self-expanding, cylindrical nature of the device makes it easy to use and to appropriately position in the aorta proximal to the innominate artery for complete cerebral protection. It is suitable for the vast majority of varied aortic arch anatomies and does not require cannulation of the head and neck vessels or multiview angiography for confirmation of device orientation or appropriate coverage. Second, the cylindrical nature of the F_2_ increases the surface area of apposition to the aortic wall and helps to achieve stability of the filter during the transit of the TAVR device. Finally, the electrospun polyurethane filtration membrane has a much lower average pore size than previously available devices at 30 μm, which may translate to improved filtration and deflection of smaller debris particles.

The current study provides preliminary evidence that the EnCompass F_2_ filter is feasible and safe for TAVR with commercially available balloon-expandable and self-expanding TAVR systems. The F_2_ device proved to be easy to use with an average deployment time of only about 3 minutes, use of only a single filter in all cases, and no significant device-device interaction or filter migration. Device technical and procedure success were achieved in 100% of cases, and there were no procedural complications, stroke, or 30-day MACCEs. Notably, F_2_ filter deployment was feasible from both contralateral vascular access in the initial cases and subsequently from ipsilateral access (42%), which has potential advantage of avoiding a second large-bore vascular access but requires externalization of the retention wire at the TAVR access site. Although there were no overt bleeding or vascular complications, the small sample size precludes meaningful comparison between these vascular access approaches. Importantly, this study did not demonstrate any clinical sequelae to suggest downstream effects of debris deflection, such as acute kidney injury or distal vascular complications, but this remains a theoretical concern of arch deflector CEPDs.

The DW-MRI results of the present study support the ability of the F_2_ to effectively isolate the head and neck vessels through apposition of the F_2_ frame to the aortic wall and filtration of the blood supply to the brain through the electrospun membrane. The median lesion number and volumes in the present study were all substantially lower than those observed in prior studies^9^^,^^10^ with the SENTINEL or TriGUARD devices (Figure 4), perhaps due to the more complete coverage and the much smaller pore size of the F_2_ filter.^9^, ^10^, ^11^, ^12^^,^^14^, ^15^, ^16^ A recent pooled analysis of almost 500 patients from the TriGUARD experience has established that DW-MRI number, size, and total volume of acute brain infarction are each associated with clinical ischemic strokes, disabling strokes, and worse stroke recovery at 30 days in patients undergoing TAVR.^17^ Further study will be therefore be required to prove that the promising results observed with the F_2_ translate into clinical differences in stroke or other neurological end points, such as cognitive decline.

An interesting feature of the present study was the use of remote neurological examination performed by telehealth by vascular neurologists at a centralized core laboratory. In the context of larger studies, this may serve to better standardize stroke and other neurological assessments through the involvement of a relatively limited number of neurologists and a more reproducible methodology. The involvement of site-level neurological professionals has also been a logistical barrier in prior studies, and remote examination may help to overcome these barriers and save resources in future trials.

### Limitations

This report describes a relatively small FIH experience, and the results should be considered hypothesis-generating only. Subjects were carefully selected, and cases were performed by a limited number of operators at a single medical center, so the generalizability of these results remains uncertain. Adequately powered, randomized, controlled, clinical trials are planned to conclusively demonstrate the safety and efficacy F_2_ filter in preventing stroke and brain injury during TAVR. Further study is also required to clarify the optimal vascular access approach (ipsilateral vs contralateral), define the limits of suitable iliofemoral and aortic arch anatomy for the F_2_ filter, and exclude any distal embolic effects of the aortic arch deflection strategy for cerebral protection.

## Conclusion

In this FIH series, cerebral embolic protection with the EnCompass F_2_ filter during transfemoral TAVR was demonstrated to be feasible and safe. DW-MRI of the brain within 72 hours demonstrated very low total new lesion numbers and volumes, and larger clinical trials are justified to confirm these findings.

## Supplementary material

Supplementary Material

Video 1A **F_2_ filter use during TAVR.** (**A**) F_2_ Filter deployment within the ascending aorta, 2cm proximal to the innominate artery using a pin (Filter Wire) and pull (F_2_ Delivery Sheath) technique. (**B**) Traversal of the transcatheter valve delivery system through the central lumen of the F_2_ device. (**C**) At the completion of the TAVR procedure, the F_2_ Delivery Sheath is reinserted over the 0.015” filter retention wire in order to re-sheath the filter for retrieval. TAVR, transcatheter aortic valve replacement.

Video 1B **F_2_ filter use during TAVR.** (**A**) F_2_ Filter deployment within the ascending aorta, 2cm proximal to the innominate artery using a pin (Filter Wire) and pull (F_2_ Delivery Sheath) technique. (**B**) Traversal of the transcatheter valve delivery system through the central lumen of the F_2_ device. (**C**) At the completion of the TAVR procedure, the F_2_ Delivery Sheath is reinserted over the 0.015” filter retention wire in order to re-sheath the filter for retrieval. TAVR, transcatheter aortic valve replacement.

Video 1C **F_2_ filter use during TAVR.** (**A**) F_2_ Filter deployment within the ascending aorta, 2cm proximal to the innominate artery using a pin (Filter Wire) and pull (F_2_ Delivery Sheath) technique. (**B**) Traversal of the transcatheter valve delivery system through the central lumen of the F_2_ device. (**C**) At the completion of the TAVR procedure, the F_2_ Delivery Sheath is reinserted over the 0.015” filter retention wire in order to re-sheath the filter for retrieval. TAVR, transcatheter aortic valve replacement.
