# Supplementary material for: First-in-Human Experience With the EnCompass F2 Filter, a Novel Cerebral Embolic Protection Device for TAVR
Source: J Soc Cardiovasc Angiogr Interv. 2025 Aug 12;4(9):103608. doi: 10.1016/j.jscai.2025.103608 (PMC12485541; doi:10.1016/j.jscai.2025.103608)
Supplement: Supplementary Material [file mmc1.docx]

Supplementary Table 1. Inclusion and Exclusion Criteria

**Inclusion criteria**

For inclusion in the study patients should fulfil the following criteria:

1. Age ≥ 22 years.
2. The patient meets the established criteria and indications for commercially available TAVR (Edwards/Medtronic/Myval) for transfemoral access.
3. Modified Rankin Scale (mRS) 0 or 1 at screening.
4. The patient is willing and able to comply with protocol- specified follow-up evaluations.
5. The patient or legally authorized representative is able and willing to provide written informed consent

**Exclusion criteria**

Patients should not enter the study if any of the following exclusion criteria are fulfilled:

1. Contraindications to MRI (e.g., participants with MR unsafe implants including implantable temporary or permanent pacemaker or defibrillator, metal implants in field of view, metallic fragments, clips, or devices in the brain or eye before TAVR procedure, or claustrophobia).
2. Severe peripheral arterial, abdominal aortic, or thoracic aortic disease that precludes delivery sheath vascular access or filter deployment.
3. Patients in whom the aortic arch is heavily calcified, severely atheromatous, or severely tortuous.
4. Ascending aortic diameter > 38 mm and transverse aortic diameter > 27 mm.
5. Evidence of an acute myocardial infarction within 1 month before TAVR.
6. Pre-existing prosthetic heart valve or prosthetic ring in any position.
7. Known intracardiac thrombus.
8. Severe allergy or known hypersensitivity or contraindication to aspirin, heparin/bivalirudin, clopidogrel, nitinol, stainless steel alloy, and/or contrast sensitivity that cannot be adequately pre-medicated.
9. History of bleeding diathesis or coagulopathy or patients in whom anti-platelet and/or anticoagulant therapy is contraindicated.
10. Patients who refuse blood transfusion.
11. Active peptic ulcer or history of upper gastrointestinal (GI) bleeding within the prior 3 months.
12. Recent (within 6 months) CVA or a TIA.
13. Renal insufficiency (creatinine > 2.5 mg/dL or GFR < 30) and/or renal replacement therapy at the time of screening.
14. Patients with hepatic failure (Child-Pugh class C).
15. Patients with hypercoagulable states that cannot be corrected by additional periprocedural heparin.
16. Patients presenting with cardiogenic shock or requiring vasopressor or inotropic support at the time of the index procedure.
17. Severe ventricular dysfunction with left ventricular ejection fraction (LVEF) < 20%.
18. Life expectancy < 12 months.
19. Currently participating in an investigational drug or another device study that has not completed the initial primary endpoint evaluation (e.g., long term follow-up study/continued access study participants are able to be included).
20. Subjects who have had treatment with any other investigational device within 30 days prior to study enrollment.
21. Planned surgery or invasive procedure within 30 days following the index procedure (TAVR with F2 Filter).
22. Patients planned to undergo any other cardiac surgical or interventional procedure (e.g., concurrent coronary revascularization) during the TAVR procedure or within 10 days prior to the TAVR procedure. *NOTE: Balloon valvuloplasty during TAVR is permitted. Diagnostic cardiac catheterization is permitted within 10 days prior to the TAVR procedure.*
23. Patients with known mental or physical illness or known history of substance abuse that may cause non-compliance with the protocol or confound the data interpretation.
24. Females who are pregnant or nursing or plan to become pregnant during their participation in the study. Female participants of child-bearing potential must have a negative pregnancy test at screening.
25. Any other condition that in the opinion of the investigator, precludes study participation or poses a significant hazard to the patient.
